# The International Space Station Environment Triggers Molecular Responses in *Aspergillus niger*

**DOI:** 10.3389/fmicb.2022.893071

**Published:** 2022-06-30

**Authors:** Adriana Blachowicz, Jillian Romsdahl, Abby J. Chiang, Sawyer Masonjones, Markus Kalkum, Jason E. Stajich, Tamas Torok, Clay C. C. Wang, Kasthuri Venkateswaran

**Affiliations:** ^1^Department of Pharmacology and Pharmaceutical Sciences, School of Pharmacy, University of Southern California, Los Angeles, CA, United States; ^2^Biotechnology and Planetary Protection Group, Jet Propulsion Laboratory, California Institute of Technology, Pasadena, CA, United States; ^3^Department of Immunology and Theranostics, Beckman Research Institute of City of Hope, Duarte, CA, United States; ^4^Department of Microbiology and Plant Pathology, Institute for Integrative Genome Biology, University of California, Riverside, Riverside, CA, United States; ^5^Ecology Department, Lawrence Berkeley National Laboratory, Berkeley, CA, United States; ^6^Department of Chemistry, Dornsife College of Letters, Arts, and Sciences, University of Southern California, Los Angeles, CA, United States

**Keywords:** *Aspergillus niger*, International Space Station, metabolome, proteome, genome

## Abstract

Due to immense phenotypic plasticity and adaptability, *Aspergillus niger* is a cosmopolitan fungus that thrives in versatile environments, including the International Space Station (ISS). This is the first report of genomic, proteomic, and metabolomic alterations observed in *A. niger* strain JSC-093350089 grown in a controlled experiment aboard the ISS. Whole-genome sequencing (WGS) revealed that ISS conditions, including microgravity and enhanced irradiation, triggered non-synonymous point mutations in specific regions, chromosomes VIII and XII of the JSC-093350089 genome when compared to the ground-grown control. Proteome analysis showed altered abundance of proteins involved in carbohydrate metabolism, stress response, and cellular amino acid and protein catabolic processes following growth aboard the ISS. Metabolome analysis further confirmed that space conditions altered molecular suite of ISS-grown *A. niger* JSC-093350089. After regrowing both strains on Earth, production of antioxidant—Pyranonigrin A was significantly induced in the ISS-flown, but not the ground control strain. In summary, the microgravity and enhanced irradiation triggered unique molecular responses in the *A. niger* JSC-093350089 suggesting adaptive responses.

## Introduction

The International Space Station (ISS) is a research facility orbiting at an approximate altitude of 250 miles that is utilized to study physiological, psychological, and immunological responses of humans living in isolation (Mehta et al., 2004; Crucian et al., 2013; Cucinotta, 2014; Benjamin et al., 2016; Ombergen et al., 2017). However, the distinct ISS environment, which includes microgravity and enhanced irradiation, affects the metabolism of all living organisms aboard the ISS including humans. There is a growing body of research that focuses on molecular characterization of animal (Ijiri, 2003; Tavella et al., 2012), plant (Link et al., 2003; Driss-Ecole et al., 2008; Kittang et al., 2014), and microbial (Rabbow et al., 2003; Benoit et al., 2006) responses to the conditions encountered in the ISS. Among the most studied microorganisms are various species of bacteria (Klaus et al., 1997; Weng et al., 1998; Nickerson et al., 2000; Vaishampayan et al., 2012), yeast (Takahashi et al., 2001; Purevdorj-Gage et al., 2006; Altenburg et al., 2008; Liu et al., 2008; Crabbé et al., 2013), and black fungi (Onofri et al., 2008, 2012, 2015). However, there are few reported studies that characterize the molecular responses of filamentous fungi (Romsdahl et al., 2018, 2019, 2020; Blachowicz et al., 2019b).

Filamentous fungi are producers of a myriad of bioactive compounds or secondary metabolites (SMs). These SMs often confer environmental advantage, which facilitate survival in hostile niches despite not being directly essential for survival (Keller et al., 2005; Fox and Howlett, 2008; Brakhage and Schroeckh, 2011; Rohlfs and Churchill, 2011; Brakhage, 2013). SMs span from potent bioactive molecules used in the drug discovery processes (Borel et al., 1995; Elander, 2003; Mulder et al., 2015) or other branches of the industry (Vandenberghe et al., 2000; Rodríguez Couto and Toca Herrera, 2006; Piscitelli et al., 2010; Dhillon et al., 2011) to health hazardous toxins (Barnes, 1970; Shephard, 2008; Hof and Kupfahl, 2009; Eaton and Groopman, 2013). Altered production of various SMs is one potential mechanism of fungal adaptation to extreme environments. For example, increased production of melanin, a pigment with UV protective properties, was observed in fungi isolated from Chernobyl nuclear power plant (Dadachova et al., 2008) and “Evolution Canyon” (Singaravelan et al., 2008). One such highly melanized fungal species is *Aspergillus niger*.

Industrially important *A. niger* (Schuster et al., 2002) has been isolated from various ecological niches, including decaying leaves (Nikolcheva et al., 2003), common households (Adams et al., 2013; Barberán et al., 2015), and the ISS (Checinska et al., 2015). The *A. niger* strain JSC-093350089 isolated from the surface of the US compartment of the ISS was previously characterized using multi-omics techniques. Performed analyses revealed genetic variance typical for the *A. niger* clade, increased abundance of proteins involved in starvation response, oxidative stress, and cell wall modulation (Romsdahl et al., 2018), and alteration in SM production levels when compared to well-studied *A. niger* ATCC 1015 strain (Romsdahl et al., 2019). However, definite ascribing of observed molecular alterations to the ISS environment was not possible, since the strains were not grown in microgravity using a controlled experiment with ground counterparts. Nevertheless, in-depth characterization of ISS-isolated JSC-093350089 *A. niger* provided insight into potential space-induced molecular phenotypes.

This study is the first report of the multi-omics characterization of *A. niger* JSC-093350089 grown aboard the ISS and compared to ground controls. To study the impact of the enhanced irradiation and microgravity on JSC-093350089, the strain was transported to and grown aboard the ISS. Upon return to Earth, ISS-grown samples, along with ground controls, were immediately processed for metabolomic, proteomic, and genomic analyses with the aim of obtaining important insights into the adaptive responses of *A. niger* to space conditions. In addition, ISS-grown samples were regrown on Earth to identify any conserved molecular alterations.

## Materials and Methods

### Isolation and Identification of *Aspergillus niger*

Procedures to isolate and identify *A. niger* collected from the ISS were described previously (Romsdahl et al., 2018). In brief, sterile swabs soaked in saline solution were used to sample the ISS surface and transported to Earth. Particles retrieved from the swab were spread into potato dextrose agar (PDA) plates and any growing colonies were purified, collected, and further analyzed. One of the collected isolates was identified as *A. niger via* ITS region sequencing, which was subsequently confirmed *via* whole-genome sequencing (WGS).

### Growth Conditions

JSC-093350089 was cultivated on glucose minimal medium (GMM) agar plates (6 g/l NaNO_3_, 0.52 g/l KCl, 0.52 g/l MgSO_4_·7H_2_O, 1.52 g/l KH_2_PO_4_, 10 g/l D-glucose, and 15 g/l agar supplemented with 1 ml/l of Hutner’s trace elements) covered with a cellophane membrane. Each of 10 prepared Petri plates (D = 10 cm) was inoculated with 1 × 10^7^ conidia/plate. Subsequently, plates were sealed with 3 M^™^ Micropore^™^ Surgical Tape (VWR International, Radnor, PA, United States) and placed in four Biological Research in Canister (BRIC) systems (three and two plates/BRIC). BRICs were divided into two groups, which were exact mimics, and transferred to 4°C. The exact timeline of the experiment is presented in Supplementary Figure 1. The whole experiment lasted 42 days from preparing the payload by a science team prior to launch till the handout of the ISS-grown samples back to the science team after the flight. The first group of BRICs was sent to the ISS and continuously kept at 4°C (1–20 days) prior to being transferred to ambient temperature for the active growth phase ~22°C for 12 days (21–32 days). After that time BRICs were stored at 4°C before returning to Earth (33–42 days). Upon arrival to Earth, BRICs were turned over to the science team for the downstream analyses, which commenced immediately. The second group of BRICs, treated as controls, was kept on Earth at Kennedy Space Center (KSC) and mimicked the ISS experiment timeline with roughly 2 h of delay. BRICs containing control samples were shipped from KSC to research team along with the ISS-grown samples. Lastly, for an additional secondary metabolite analysis, 1 × 10^7^ conidia/plate of the ISS- and ground-grown JSC-093350089 were grown on GMM medium at 28°C for 5 days.

### Genomic DNA Extraction and Whole-Genome Sequencing

Mycelia and conidia were collected from ground-and ISS-grown JSC-09335008 GMM agar plates. DNA was extracted using the Power Soil DNA Isolation Kit (Mo Bio Laboratories, Carlsbad, California, United States) following the manufacturers protocol. Extracted DNA was checked for quality using Qubit 2.0 Fluorometer and used for paired-end library preparation with TruSeq Nano DNA Library Preparation Kit (Illumina, San Diego, California, United States) followed by WGS at the Duke Center for Genomic and Computational Biology. Samples were sequenced using a HiSeq 4,000 Illumina Sequencer generating 101 base long reads.

### Genetic Variation Identification

Illumina sequence reads were trimmed using Trimmomatic v 0.36 (Bolger et al., 2014) and checked for quality using FastQC v 0.11.5 (Andrews, 2010). The genome and annotation files for *A. niger* CBS 513.88, (Pel et al., 2007) were downloaded from the FungiDB web portal (Stajich et al., 2012). Reads were mapped to CBS 513.88 the reference genome using the Burrows-Wheeler Aligner (BWA) software package v 0.7.12 (Li and Durbin, 2009) and further processed with SAMtools v 1.6 to generate sorted BAM files (Li et al., 2009). SNPs and INDELs were identified using GATK v 3.7 (DePristo et al., 2011). Duplicates were marked using Picard-tools MarkDuplicates^1^ to remove PCR artifacts. Sequence reads containing putative INDELs were realigned using GATK’s IndelRealigner to generate an updated BAM file. Variants within each sample were called using GATK’s Haplotype Caller. GATK’s VariantFiltration was used to filter each VCF file based on stringent cutoffs for quality and coverage {SNPs: QD < 2.0, MQ < 40.0, QUAL < 100, FS > 60.0, MQRankSum < −12.5, SOR > 4.0, ReadPosRankSum < −8.0; Indels: QD < 2.0, FS > 200.0, MQRankSum < −12.5, SOR > 4, InbreedingCoeff < −0.8, ReadPosRankSum < −20.0}, so that only high-quality variants remained.

### Protein Extraction

Mycelia and conidia from GMM agar plates were collected and stored at –80°C prior to protein extraction. Proteins were extracted with the lysis buffer consisting of 100 mm triethylammonium bicarbonate (TEAB) with 1:100 Halt Protease Inhibitor Cocktail (Thermo Scientific, Rockford, IL) and 200 μg/ml phenylmethylsulfonyl fluoride (Sigma-Aldrich, St. Louis, MO, United States). Mycelia and conidia were homogenized by bead beating using Precellys 24 homogenizer (Bertin, Rockville, MD). The lysed fungal material was centrifuged at 17,000 g for 15 min and the protein concentration in the supernatants was measured by the Bradford assay (Bio-Rad Laboratories, Inc. Hercules, CA, United States).

### Tandem Mass Tag (TMT) Labeling

A 100 μg proteins from each sample were precipitated in 20% trichloroacetic acid (TCA) at 4°C. Protein pellets were washed with ice-cold acetone and re-suspended in 25 μl TEAB (100 mM) and 25 μl 2,2,2-trifluoroethanol (TFE). Proteins were reduced with 1 μl of tris(2-carboxyethyl)phosphine (TCEP, 500 mM), alkylated with iodoacetamide (IAA, 30 mM), and digested with 2.5 μg/sample of trypsin/lysC (Promega, Madison, WI, United States) overnight at 37°C. The digested peptides were quantified using the Pierce Quantitative Colorimetric Peptide Assay (Thermo Scientific, Waltham, MA, United States). 40 μg of peptides from each specific sample was labeled with the Thermo Scientific TMTsixplex Isobaric Mass Tagging Kit (JSC-E1 (ground 1) with TMT^6^-128, JSC-E2 (ground 2) with TMT^6^-130, JSC-S1 (ISS 1) with TMT^6^-129, JSC-S2 (ISS 2) with TMT^6^-131) according to the manufacturer’s protocol. All labeled-peptide mixtures were combined into a single tube, mixed, and fractionated using the Thermo Scientific Pierce High pH Reversed-Phase Peptide Fractionation Kit. While this kit usually uses eight fractions with step elution of up to 50% acetonitrile, ninth fraction was added eluting at 100% acetonitrile. Nine fractionated samples were dried using a SpeedVac concentrator and re-suspended in 1% (v/v) formic acid prior to liquid chromatography with tandem mass spectrometry (LC-MS/MS) analysis.

### LC–MS/MS Analysis

The samples were analyzed on an Orbitrap Fusion Tribrid mass spectrometer with an EASY-nLC 1,000 Liquid Chromatograph, a 75 μm × 2 cm Acclaim PepMap100 C18 trapping column, and a 75 μm × 25 cm PepMap RSLC C18 analytical column, and an Easy-Spray ion source (Thermo Scientific, Waltham, MA, United States). The peptides were eluted at 45°C with a flow rate of 300 nl/min over a 110 min gradient, from 3 to 30% solvent B (100 min), 30–50% solvent B (3 min), 50–90% solvent B (2 min), and 90% solvent B (2 min). The solvent A was 0.1% formic acid in water and the solvent B was 0.1% formic acid in acetonitrile.

The full MS survey scan (m/z 400–1,500) was acquired at a resolution of 120,000 and an automatic gain control (AGC) target of 2 × 10^5^ in the Orbitrap with the 50 ms maximum injection time for MS scans. Monoisotopic precursor ions were selected with charge states 2–7, a ± 10 ppm mass window, and 70 s dynamic exclusion. The MS^2^ scan (m/z 400–2000) was performed using the linear ion trap with the 35% collision-induced dissociation (CID) energy. The ion trap scan rate was set to “rapid,” with an AGC target of 4 × 10^3^, and a 150 ms maximum injection time. Ten fragment ions from each MS^2^ experiment were subsequently selected for an MS^3^ experiment. The MS^3^ scan (m/z 100–500) was performed to generate the TMT reporter ions in the linear ion trap using higher-energy collisional dissociation (HCD) at a 55% collision energy, a rapid scan rate and an AGC target of 5 × 10^3^, and a maximum injection time of 250 ms.

### Proteome Data Analysis

All MS data (MS^1^, MS^2^, and MS^3^) were searched using the Proteome Discoverer (version 2.2.0.388, Thermo Scientific) with the Sequest-HT searching engines against an *Aspergillus niger* CBS 513.88 database containing 10,549 sequences (NCBI). The searches were performed with the following parameters: 2 maximum missed cleavage sites, 6 minimum peptide length, 5 ppm tolerance for precursor ion masses, and 0.6 Dalton tolerance for fragment ion masses. The static modification settings included carbamidomethyl of cysteine residues, and dynamic modifications included oxidation of methionine, TMT6plex modification of lysine ε-amino groups and peptide N-termini, and acetyl modification of protein N-terminus. A false discovery rate (FDR) of 1% was set using a target-decoy database search. The reporter ions integration tolerance was 0.5 Da and the co-isolation threshold was 75%. The average signal-to-noise threshold of all reporter peaks was bigger than 10. The total intensity of a reporter ion for a protein was calculated based on the sum of all detected reporter ions of associated peptides from that protein. The ratios between an abundance of a reporter and the average abundance of all reporters were used to estimate the abundance ratio of each protein.

For statistical analysis, the sum of reporter ion intensities for each protein was Log2 transformed and the biological duplicate and technical triplicate measurement for each protein was averaged. Only the proteins that were identified and quantified in both biological and technical replicates were used for the analysis. Student t-test was performed to identify proteins with changed abundance. Proteins with value of *p* <0.05 were further evaluated for increased and decreased abundance using a cut-off value of ≥ ± 1 fold (Log2) change.

### Secondary Metabolite Extraction and Analysis

Upon return to Earth single plates from both ground- and ISS-kept BRICs were used to collect agar plugs in triplicate for secondary metabolite (SM) extraction. Plugs were extracted with 3 ml of methanol and 1:1 methanol/dichloromethane each followed by 1 h sonication. Crude extract was evaporated *in vacuo* to yield a residue that was then suspended in 1 ml of 20% dimethyl sulfoxide/methanol and 10 μl was examined by high-performance liquid chromatography-photodiode array detection-mass spectrometry (HPLC-DAD-MS) analysis. HPLC–MS was carried out using ThermoFinnigan LCQ Advantage ion trap mass spectrometer with an RP C18 column (Alltech Prevail C18 3 mm 2.1 × 100 mm) at a flow rate 125 μl/min. The solvent gradient for LC–MS was 95% acetonitrile/H2O (solvent B) in 5% acetonitrile/H2O (solvent A) both containing 0.05% formic acid, as follows: 0% solvent B from 0 min to 5 min, 0 to 100% solvent B from 5 min to 35 min, 100% solvent B from 35 min to 40 min, 100 to 0% solvent B from 40 min to 45 min, and re-equilibration with 0% solvent B from 45 min to 50 min. The SM profiles of ISS- and ground-grown JSC-093350089, which were regrown at 28°C were obtained following the procedure described above.

### SM Statistical Analysis

To compare the yields of produced SMs in ISS-grown, ground-grown, and regrown samples, the area under the electrospray ionization curve (ESI) was integrated for each compound. SM data collected from three independent biological replicates of ISS- and ground-grown, and regrown JSC-093350089 were used for testing statistical significance of production yields of identified SMs by Welch’s corrected t-test. The data are presented as column charts with corresponding error bars. Data analysis was conducted using GraphPad Prism version 7.

## Results

### Genome Variation in the ISS-Grown JSC-093350089 *Aspergillus niger*

The genomes of ISS-flown and ground-grown JSC-093350089 were compared to identify occurring genetic variations. Obtained reads were aligned to the CBS 513.88 reference genome and single-nucleotide polymorphisms (SNPs) present in the ground control were filtered. This revealed presence of 375 SNPs and 620 INDELs that occurred because of the exposure to conditions aboard the ISS (Table 1). All identified genetic variations are summarized in Supplementary Tables 1 and 2, presenting SNPs and INDELs, respectively. Distribution of non-synonymous point mutations among genes is presented in Table 2. Interestingly, about 80% of these mutations occurred in chromosome VIII and 13% occurred in chromosome XII, while the remaining 7% were distributed among other chromosomes (Supplementary Figure 2A). The majority of missense point mutations (75%) were observed within genes of unknown function. However, several characterized genes containing missense SNPs have DNA-binding activity (An06g01180, An08g11890, and An12g00840), DNA polymerase activity (An08g11520), protein kinase and transferase activity (An08g12110), phospholipase activity (An08g12250), and chromosome anchoring RacA protein binding activity (An12g06420; Table 2). Additional, mutations like one stop lost, one start gained and one 5 prime untranslated region (UTR) mutation were observed. Most of the observed SNPs (~55%) and INDELs (71%) were located in intergenic regions (Table 1). Interestingly, unlike SNPs, INDELs were distributed throughout all chromosomes. However, similarly to SNPs, the highest number of INDELS was found in chromosomes VIII and XII. Among observed INDELS 109 caused frameshift, 14 lead to disruptive inframe deletion, and a few caused start lost and stop gained (Supplementary Table 2; Supplementary Figure 2B).

**Table 1:** Summary of genetic variations observed in ISS-grown JSC-093350089 when compared to ground control.

	**Type of mutation**	**Occurrence number**
**SNPs**	Intergenic	205
	Missense	79
	Splice region	4
	Start gained	1
	Stop lost	1
	5 prime UTR	1
	Synonymous	84
**Total no. of SNPs**		375
**INDELs**	Intergenic	444
	5 prime UTR	10
	Conservative inframe deletion	8
	Disruptive inframe deletion	14
	Frameshift	109
	3 prime UTR	15
	Splice region	11
	Start lost	1
	Stop gained	8
**Total no. of INDELs**		620

**Table 2:** Single-nucleotide polymorphisms (SNPs) in ISS-grown JSC-093350089 when compared to ground control.

**Function**	**Gene**	**Base mutation when compared to ground control**	**Type of mutation**
RNA polymerase II transcription factor activity, sequence-specific DNA binding	An06g01180	An06_G279214A	5 prime UTR
RNA-directed DNA polymerase activity and role in RNA-dependent DNA replication	An08g11520	An08_G2725926T	Missense
		An08_T2726169G	
		An08_C2726328A	
		An08_T2726340C	
		An08_A2726541G	
		An08_A2726556G	
		An08_C2726566A	
		An08_G2726574T	
		An08_G2726997A	
		An08_C2727012T	
		An08_G2728798A	
		An08_C2729323T	
		An08_G2726128T	Stop lost
DNA-binding activity	An08g11890	An08_A2824556C	Missense
		An08_C2824566G	
Protein kinase and transferase activity	An08g12110	An08_G2869126A	Missense
		An08_T2869564C	
Phospholipase	An08g12250	An08_C2920251G	Missense
		An08_A2920254C	
		An08_C2920298T	
		An08_A2920340G	
DNA binding, RNA polymerase II transcription factor activity	An12g00840	An12_T222942C	Missense
		An12_G222974T	
		An12_G222982A	
		An12_G223213A	
		An12_G223422C	
		An12_G223434A	
		An12_A223471G	
		An12_G222400A	Splice region
RacA binding protein, polarized cell growth	An12g06420	An12_A1548559G	Missense
		An12_G1548931A	
		An12_A1548987G	
		An12_A1549053C	
Unknown function	An08g08380	An08_C1997243T	Missense
Unknown function	An08g11220	An08_A2664300G	Missense
		An08_C2664502A	
Unknown function	An08g11230	An08_G2666099A	Splice region
		An08_T2666106C	
Unknown function	An08g11540	An08_A2731686T	Missense
		An08_T2731695C	
		An08_T2733283C	
		An08_C2733499T	
Unknown function	An08g11550	An08_G2734928C	Missense
Unknown function	An08g11570	An08_C2737667T	Missense
Unknown function	An08g11650	An08_G2765994T	Missense
		An08_G2766002T	
Unknown function	An08g11670	An08_G2768423A	Missense
Unknown function	An08g11830	An08_T2814800G	Missense
Unknown function	An08g11840	An08_T2817189C	Missense
		An08_A2817202G	
Unknown function	An08g11860	An08_G2818780C	Missense
		An08_G2819091A	
		An08_T2819644G	
Unknown function	An08g11870	An08_G2820964A	Missense
		An08_C2821036T	
		An08_C2821061T	
		An08_T2821094C	
		An08_A2821104C	
		An08_C2821119A	
		An08_T2821648G	
		An08_C2821664T	
		An08_T2821693A	
		An08_G2821697A	
		An08_A2821958T	
		An08_G2821978A	
		An08_T2822640C	
		An08_G2821995A	
Unknown function	An08g11880	An08_G2824016C	Missense
Unknown function	An08g11910	An08_C2827842A	Missense
		An08_C2828992G	
Unknown function	An08g11940	An08_A2835378G	Missense
Unknown function	An08g11950	An08_T2836585A	Missense
		An08_T2836586C	
		An08_A2837163G	
Unknown function	An08g11960	An08_A2839341G	Missense
		An08_G2839738A	
Unknown function	An08g12230	An08_C2911588T	Missense
		An08_T2911676C	
		An08_G2912446C	
		An08_T2912451G	
		An08_A2913614T	
		An08_T2911894A	Splice region
Unknown function	An08g12230	An08_G2913874C	Start gained
Unknown function	An08g12240	An08_A2916240C	Missense
Unknown function	An12g05800	An12_T1429831C	Missense

### Proteomic Characterization of ISS-Grown JSC-093350089 *Aspergillus niger*

**Figure 1:**
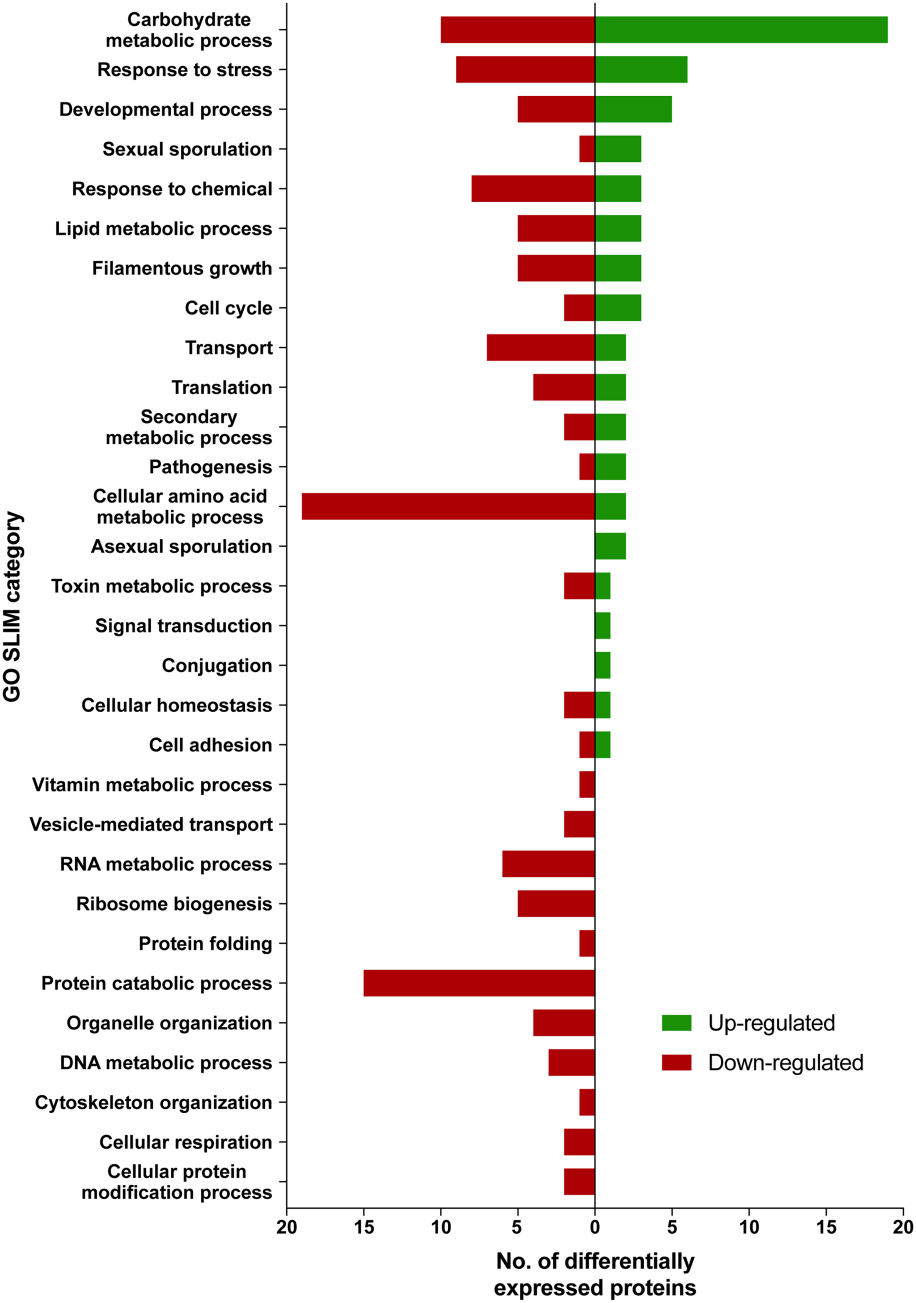
AspGD GO Slim terms of differentially expressed proteins in ISS-grown JSC-093350089. Differentially expressed proteins in FC > |2|, *p* < 0.05 were mapped to terms representing various biological processes using AspGD Gene Ontology (GO) Slim Mapper.

Differentially expressed proteins in ISS-grown JSC-093350089 strain were investigated following the extraction of total protein from two biological replicates of ISS-grown and ground control counterpart strains. Due to the low yields of extracted proteins, biological replicates were combined and divided into two parts that were then TMT labeled and subtracted to analysis *via* LC–MS/MS followed by spectrum/sequence matching using *A. niger* CBS 513.88 protein database (NCBI). Protein abundance ratios in ISS-grown JSC-093350089 were normalized to Earth-grown counterparts, which enabled identification of 70 up- and 142 downregulated proteins (fold change (FC) > ∣2∣, *p* < 0.05) in response to space conditions (Supplementary Tables 3 and 4, respectively). AspGD Gene Ontology (GO) Slim terms (Cerqueira et al., 2014) were used to study the distribution of differentially expressed proteins in ISS-grown JSC-093350089 when compared to ground controls (Figure 1). Among differentially expressed proteins, 29 were involved in carbohydrate metabolism, 18 in stress responses, 21 in amino acid metabolism, and 15 in protein catabolic processes (Figure 1). Protein GO term enrichment analysis was conducted using FungiDB (Stajich et al., 2012), which revealed that significantly over-represented upregulated biological processes included carbohydrate metabolic processes (28% of all upregulated proteins) and stress response (10%), whereas significantly over-represented downregulated processes included cellular amino acid metabolic processes (13%), proteasomal ubiquitin-independent (10%) and dependent processes (10%), and proteasomal protein catabolic processes (10%; Supplementary Table 5).

The majority of differentially expressed proteins in ISS-grown JSC-093350089 *A. niger* were involved in carbohydrate metabolism (Table 3). Interestingly, eight of these genes, including cellobiohydrolases A and B (An07g09330 and An01g11660), XlnA 1,4-β-xylanase (An03g00940), and D-xylose reductases XyrA and XdhA (An01g03740 and An12g00030) were regulated by XlnR. XlnR is a transcriptional regulator involved in degradation of polysaccharides, xylan, cellulose, and D-xylose (Hasper et al., 2000). β-glucanases An11g01540 and An02g00850, which are involved in carbon starvation response in *A. niger* (Nitsche et al., 2012), were at minimum 3-fold upregulated in ISS-grown strain. α-1,2-mannosidases An08g08370, An13g01260 were at least 3.5-fold upregulated, whereas pyruvate decarboxylase PdcA (An02g06820) was nearly 3-fold upregulated. Pyruvate kinase PkiA (An07g08990), pyruvate dehydrogenase Pda1 (An07g09530), and isocitrate lyase AcuD (An01g09270) were at least 2-fold less abundant in ISS-grown samples. Several proteins involved in the stress response were differentially expressed in ISS-grown JSC-093350089 (Table 4). Proteins exhibiting at least 2-fold upregulation included cell wall organization protein EcmA (An04g01230) and An16g07920, whose orthologs play a role in salt stress response. Downregulated stress response proteins included heat shock protein An06g01610, DNA-binding protein HttA (An11g11300), and quinone reductase An12g06300. Lastly, a variety of proteins involved in cellular amino acid processes (Table 5), and protein catabolic processes (Table 6) were downregulated.

**Table 3:** Differentially expressed proteins involved in carbohydrate metabolism.

**ORF**	**Protein**	**CAZy Family**	**Function / Activity**	**Relative protein abundance** ^*^	***p*-value**
An03g00940	XlnA/XynA	GH10	1,4-β-xylanase	2.22	2.66E-03
An01g11660	CbhB	GH7, CBM1	Cellobiohydrolase B	2.00	2.49E-03
An03g00500		GH30	1,6-β-glucosidase	1.97	6.91E-03
An11g01540		GH16	β-glucanase	1.93	4.20E-03
An08g08370		GH92	α-1,2-mannosidase	1.93	5.22E-03
An13g01260		GH92	α-1,2-mannosidase	1.83	4.16E-03
An15g04900		AA9, CBM1	β-1,4-glucanase D	1.77	2.18E-02
An11g03340	AamA	GH13	acid α-amylase	1.71	8.95E-04
An02g00850		GH16	β-glucanase	1.70	4.28E-02
An11g01120	Alr	–	Erythrose reductase	1.65	7.94E-04
An15g07800	AgtC	GH13	4-α-glucanotransferase	1.61	2.64E-03
An03g00960	AxhA	GH62	α-L-arabinofuranosidase	1.60	9.66E-03
An02g06820	PdcA	–	Pyruvate decarboxylase	1.57	5.76E-03
An02g11150	AglB	GH27	α-galactosidase II	1.37	2.89E-03
An08g01710	AbfC	GH51	Arabinofuranosidase	1.36	4.94E-04
An14g02760	EglA	GH12	β-1,4-glucanase	1.27	6.70E-05
An14g02070		CEnc	Acetylxylan esterase	1.27	8.67E-03
An05g02410		GH2	Glycoside hydrolase	1.10	1.77E-02
An07g09330	CbhA	GH7	Cellobiohydrolase A	1.07	1.44E-03
An01g03740	XyrA	–	D-xylose reductase	−1.08	2.59E-02
An12g00030	XdhA	–	D-xylulose reductase	−1.10	1.72E-02
An07g08990	PkiA	–	Pyruvate kinase	−1.12	2.92E-03
An18g06500		–	Phosphomannomutase	−1.14	6.84E-03
An12g03070	GlaB	GH15	Glucoamylase	−1.38	7.87E-03
An11g02550		–	Phosphoenolpyruvate carboxykinase	−1.51	3.44E-03
An15g01920	McsA	–	2-Methylcitrate synthase	−1.55	2.18E-02
An01g09270	AcuD	–	Isocitrate lyase	−1.59	9.83E-03
An15g03550		GH43	Hydrolase	−1.60	9.18E-04
An07g09530	Pda1	–	Pyruvate dehydrogenase	−2.19	2.00E-03

* *Log2 fold change of ISS-grown JSC-093350089 compared to Earth-grown counterpart (p < 0.05).*

**Table 4:** Differentially expressed proteins involved in stress response.

**ORF**	**Protein**	**Function / Activity**	Relative protein abundance*	***p*-value**
An01g14960		Asparaginase	2.21	6.46E-04
An14g02460	FhbA	Flavohemoglobin	1.56	1.45E-03
An01g02320	RasA	Ras GTPase	1.21	8.76E-03
An07g04620		Hypothetical protein	1.11	5.81E-03
An16g07920		Hypothetical protein	1.08	2.87E-02
An04g01230	EcmA	Cell wall organization protein	1.02	8.38E-04
An04g04130		Ornithine transaminase	−1.03	1.31E-02
An16g07110	Ach1	Acetyl-CoA hydrolase	−1.06	2.25E-02
An04g04870		Superoxide dismutase	−1.08	4.71E-03
An01g03740	XyrA	D-xylose reductase	−1.08	2.59E-02
An07g08990	PkiA	Pyruvate kinase	−1.12	2.92E-03
An08g00970	Rps28	Ribosomal protein of the small subunit	−1.16	4.09E-03
An02g07210	PepE	Acid aspartic protease	−1.21	1.27E-04
An12g06300		Quinone reductase	−1.25	3.28E-02
An11g11300	HttA	DNA-binding activity and role in DNA repair	−1.30	2.81E-03
An09g05870	Ndk1	Nucleoside-diphosphate kinase	−1.53	5.79E-04
An02g06560		Glutathione peroxidase / transferase activity	−1.69	9.56E-03
An06g01610		Heat shock protein	−2.08	5.35E-03

* *Log2 fold change of ISS-grown JSC-093350089 compared to Earth-grown counterpart (p < 0.05).*

**Table 5:** Differentially expressed proteins involved in cellular amino acid metabolic process.

**ORF**	**Protein**	**Function / Activity**	Relative protein abundance*	***p*-value**
An01g14960		Asparaginase	2.21	6.46E-04
An12g00160		Malate dehydrogenase	1.27	2.72E-03
An14g06010		Chorismate mutase	−1.01	1.50E-02
An04g04130		Ornithine transaminase	−1.03	1.31E-02
An16g02970		Glycine / Serine hydroxymethyltransferase	−1.05	2.74E-04
An11g09510		Aspartate semialdehyde dehydrogenase	−1.06	2.35E-03
An01g06530		Branched-chain-amino acid transaminase activity	−1.18	2.49E-02
An15g05770		Hydrogen sulfide / sulfur amino acid biosynthetic process	−1.19	1.43E-03
An15g00610		Imidazoleglycerol-phosphate dehydratase	−1.19	1.40E-03
An05g00410		Glycine/Serine hydroxymethyltransferase	−1.22	8.63E-03
An13g00550		3-Deoxy-7-phosphoheptulonate synthase	−1.36	6.77E-03
An09g03940	Ilv2	Ketol-acid reductoisomerase	−1.40	1.08E-03
An02g10750		Cysteine synthase	−1.45	3.84E-03
An11g02170		Fumarylacetoacetate hydrolase	−1.47	1.64E-03
An15g02490		Homoisocitrate dehydrogenase activity, role in lysine biosynthesis	−1.48	8.47E-03
An02g07250	AgaA	Arginase	−1.54	2.86E-03
An02g12430	IcdA	Isocitrate dehydrogenase	−1.59	1.38E-03
An08g01960		Adenosylhomocysteinase	−1.62	4.43E-03
An17g00910		Gamma-aminobutyrate transaminase	−1.72	5.69E-03
An11g02160		Maleylacetoacetate isomerase	−1.83	4.49E-03
An03g04280		Protein with similarity to pyridoxine synthesis component pyroA of *Aspergillus nidulans*	−1.88	9.72E-03

* *Log2 fold change of ISS-grown JSC-093350089 compared to Earth-grown counterpart (p < 0.05).*

**Table 6:** Differentially expressed proteins involved in protein catabolic process.

**ORF**	**Protein**	**Function / Activity**	Relative protein abundance*	***p*-value**
An02g07210	PepE	Acid aspartic protease	−1.21	1.27E-04
An18g06800	Pre10	20S CP alpha subunit of the proteasome	−1.27	1.08E-03
An04g01800		Hypothetical protein	−1.53	2.50E-03
An02g07040	Scl1	20S CP alpha subunit of the proteasome	−1.84	4.37E-03
An02g03400	Pup2	20S CP alpha subunit of the proteasome	−1.84	5.62E-03
An11g04620		Endopeptidase	−1.95	1.20E-02
An07g02010	Pre8	20S CP alpha subunit of the proteasome	−1.99	2.83E-03
An11g06720	Pre9	20S CP alpha subunit of the proteasome	−1.99	9.48E-03
An18g06680		Role in proteasomal ubiquitin-independent protein catabolic process	−2.03	4.65E-03
An04g01870		Endopeptidase activator activity	−2.05	5.99E-03
An18g06700	Pre7	20S CP beta subunit of the proteasome	−2.08	2.59E-03
An11g01760		Protein similar to proteasome 20S subunit Pre2p	−2.10	9.96E-04
An13g01210		Endopeptidase	−2.12	4.55E-03
An15g00510	Pre5	20S CP alpha subunit of the proteasome	−2.13	3.01E-03
An02g10790	Pre6	20S CP alpha subunit of the proteasome	−2.18	6.04E-03

* *Log2 fold change of ISS-grown JSC-093350089 compared to Earth-grown counterpart (p < 0.05)*.

### Metabolomic Characterization of ISS-Grown JSC-093350089 *Aspergillus niger*

**Figure 2:**
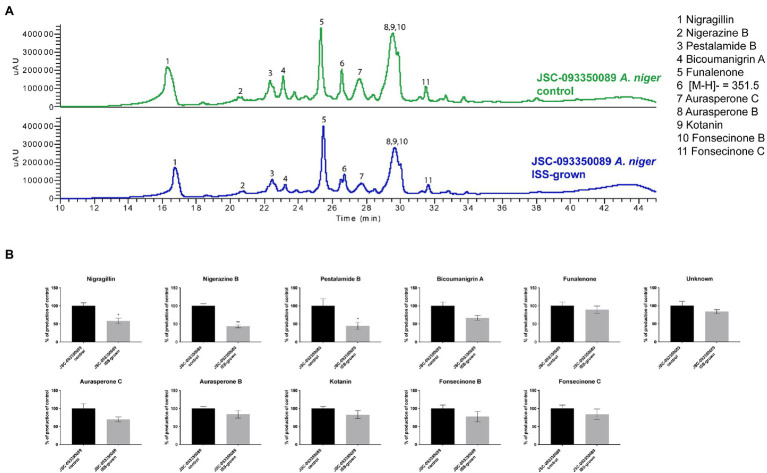
Secondary metabolite production of ISS-grown JSC-093350089 when compared to ground controls. **(A)** Secondary metabolite profiles of ISS- and ground-grown JSC-093350089 when grown on GMM. **(B)** Metabolite quantification showing the percent change for each metabolite in relation to ground-grown JSC-093350089; significance was determined using Welch’s t-test. “*” means and indicates statistical significance.

Alterations in SM production in response to ISS conditions were assessed by extracting organic compounds from three biological replicates of ISS- and ground-grown JSC-093350089. The extracts were analyzed using HPLC-DAD-MS. Compounds were identified based on mass, UV absorption, and retention time, which were in agreement with literature (Chiang et al., 2011; Figure 2). Assessment of SM production yields revealed slight decrease in production of bicoumanigrin A, fonsecinones B and C, aurasperones C and B, and kotanin in ISS-grown isolate. Production levels of pestalamide B, nigerazine B, and nigragillin exhibited a reduction of approximately 50% when compared to control strains.

**Figure 3:**
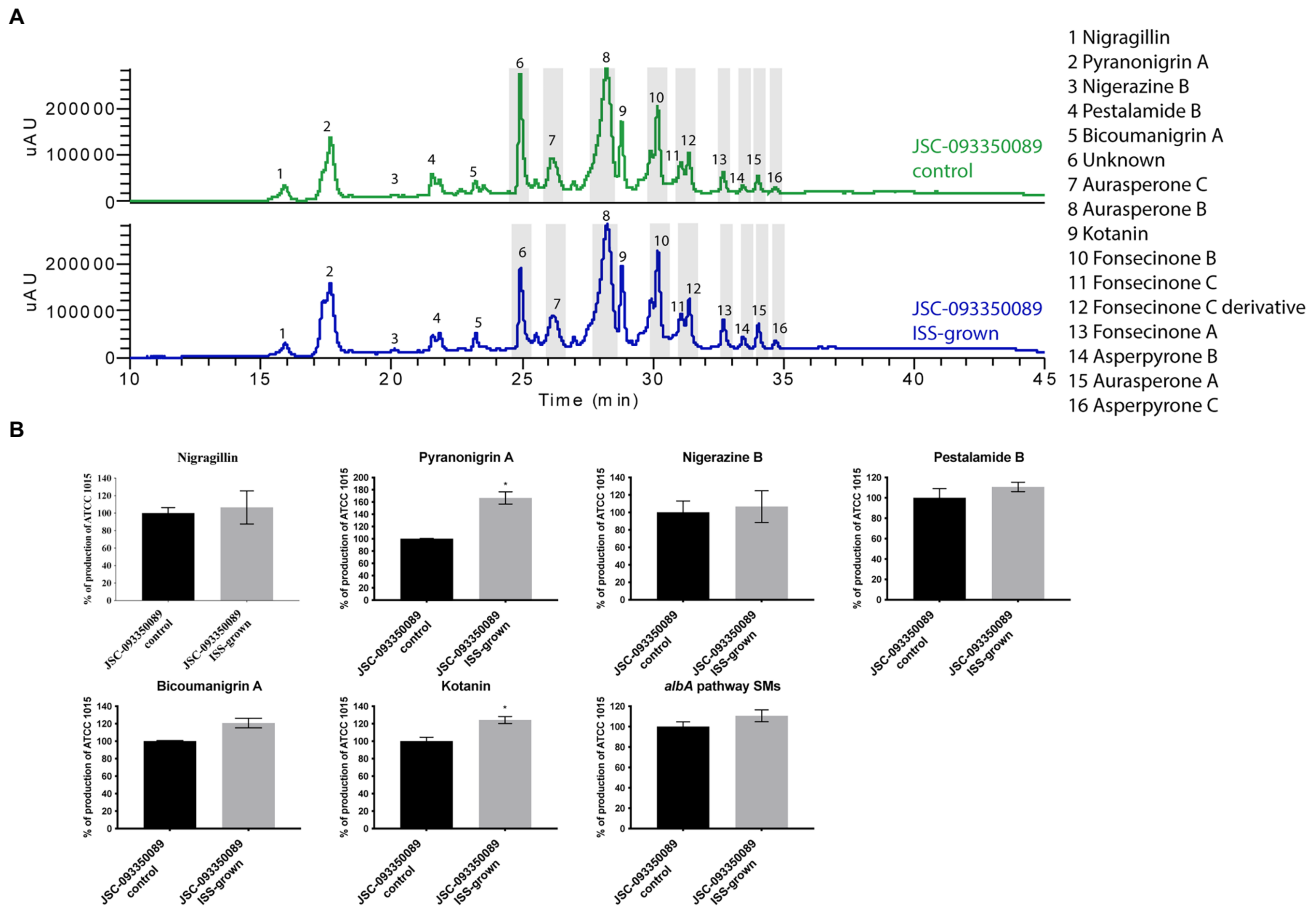
Secondary metabolite production of regrown ISS- and ground-grown JSC-093350089. **(A)** Secondary metabolite profiles of regrown ISS- and ground-grown JSC-093350089 when grown on GMM. **(B)** Metabolite quantification showing the percent change for each metabolite in relation to regrown ground-grown JSC-093350089; significance was determined using Welch’s t-test. “*” means and indicates statistical significance.

Both ISS- and ground-grown JSC-093350089 were regrown at 28°C and SMs production was evaluated. Due to the difference in growth temperature, obtained SM profiles of regrown isolates differed from those grown on the ISS (~22°C; Figure 3), as the trend in the production of SMs was the opposite of the one observed immediately after growth on the ISS. The majority of identified compounds showed slight increase in the production levels. Further, statistically significant increase in the production of the antioxidant pyranonigrin A (Miyake et al., 2007) and kotanin was observed when compared to ground controls.

## Discussion

It is critical to study molecular changes occurring in living organisms to understand the adaptation mechanisms allowing for surviving in extreme environments. One of such scientifically intriguing environments is the ISS, which is characterized by the presence of enhanced irradiation and microgravity. Due to its uniqueness, the ISS is under constant microbial monitoring, which allows for the isolation of wide array of microorganisms that often-become subjects of scientific investigations (Checinska et al., 2015; Knox et al., 2016; Checinska Sielaff et al., 2017; Venkateswaran et al., 2017; Bijlani et al., 2021). However, like in the case of the *A. niger* strain JSC-093350089 (Romsdahl et al., 2018, 2020), these investigations are more of a descriptive nature as definitive ascribing of observed molecular changes requires precisely controlled experiments. Therefore, to further investigate the differences in JSC-093350089 that were observed when compared to a “terrestrial” strain, the isolate was sent to the ISS in a planned experiment. Genomic, proteomic, and metabolomic alterations occurring in ISS-grown samples were analyzed following sample return and compared to ground-grown counterparts.

Genome analysis of ISS-grown JSC-093350089 revealed the introduction of SNPs and INDELs in response to space conditions. Interestingly, the majority of observed non-synonymous SNPs and INDELS were located within chromosomes VIII and XII, which suggests that only selected regions of the genome undergo positive selection to confer selective advantage while adapting to the space environment. This is in agreement with previous reports of space-induced genetic variations, as ISS-grown *Aspergillus nidulans* (Romsdahl et al., 2019) and spaceflight-grown *Staphylococcus aureus* (Guo et al., 2015) both exhibited genetic mutations that occurred in specific clustered regions of the genome. Although the functions of many genes containing non-synonymous SNPs were unknown, several of these genes possessed transposable element and DNA-binding activity. One such gene, An08g11520, was an analogue of transposon I factor and has RNA-directed DNA polymerase activity, which is consistent with genetic changes observed in transposable element genes in both *A. nidulans* (Romsdahl et al., 2019) and *S. aureus* (Guo et al., 2015). Alterations in transposable element genes likely influence their activity and lead to the introduction of variations within the genome in response to environmental stress (Capy et al., 2000; Muszewska et al., 2017). The results from this study further underscore the significant role of transposable elements in adaptation to the spacecraft environment. Future studies should investigate the functions of uncharacterized genes containing non-synonymous SNPs, as such knowledge may provide key information on how fungi adapt to space conditions. Noteworthy, when radioadapted strain of *Exophiala dermatitidis* and the non-radioadapted control strain were exposed to Polonium-210, a mostly transcriptomic rather than genomic response to radiation was observed in radioadapted strain. This suggests that strains previously exposed to irradiation respond to subsequent exposures in a unique way (Malo et al., 2021). Based on the observations reported for *E. dermatitidis*, it is plausible that *A. niger* JSC-093350089 strain response to the ISS environment was also unique, as it was previously isolated from the ISS and likely radioadapted itself. However, such assumption may not be confirmed in the current study, as there is no available not-radioadapted control for the *A. niger* JSC-093350089 strain. Future studies should be warranted to investigate whether *A. niger* JSC-093350089 strain’s response to the ISS environment changes with consecutive exposures to the ISS environment when compared to the *original* ISS isolate. Finally, it has been previously reported that in *Aspergillus* genome intragenic regions contain high-level developmental and metabolic transcriptional regulators (Noble and Andrianopoulos, 2013; Lim et al., 2021). Therefore, the high occurrence of SNPs and INDELS in the intergenic regions may contribute to developing the space environment-induced phenotype in *A. niger* JSC-093350089.

Investigation into the proteome of ISS- and ground-grown JSC-093350089 revealed that space conditions induce changes in protein expression when compared to ground controls. Observed, alterations in proteins involved in carbohydrate metabolism upon exposure to space conditions stay in agreement with previous studies of *A. niger* JSC-093350089 (Romsdahl et al., 2018), and other filamentous fungi, including *Aspergillus fumigatus* (Blachowicz et al., 2019a,b) and *A. nidulans* (Romsdahl et al., 2019). Similarly, a black fungus *Knufia chersonesos* exposed to low-shear simulated microgravity (LSSMG) also exhibited differential expression of proteins involved in carbohydrate metabolism (Tesei et al., 2021). On the other hand, a yeast, *Candida albicans,* exposed to spaceflight environment on the “SJ-10” satellite for 12 days showed decreased abundance of proteins involved in carbon metabolism, suggesting that yeast and filamentous fungi differ in adaptive responses to space environment (Wang et al., 2020). Additionally, ISS-grown JSC-093350089 exhibited upregulation of starvation-induced glycoside hydrolases, including AamA, and CbhB. This further confirms that changes in carbohydrate metabolism, may be a result of adaptation to scarce nutrient availability on ISS surfaces, due to the maintained cleaning regimen. Further, differential expression of XlnR-regulated proteins appears to be a key adaptive response to microgravity and enhanced irradiation, as both this and the previous study characterizing the space-induced molecular phenotype of JSC-093350089 (Romsdahl et al., 2018) revealed differential expression of various XlnR-regulated proteins. Activation of XlnR-regulated metabolic pathways leads to use of versatile carbon sources (Hasper et al., 2000), which may facilitate survival in hostile space environments. A few proteins upregulated in ISS-grown JSC-093350089 were involved in stress response, including the cell wall organization protein EcmA and flavohemoglobin, whose analogue in *Aspergillus flavus* has been reported to have a function correlated with hyphal growth phenotype (te Biesebeke et al., 2010). These observations further suggest that modulation of fungal growth and cell organization is an essential response to space conditions.

Metabolomic analysis of ISS-grown JSC-09335008 showed alterations in secondary metabolite production in response to the space environment. This observation was not surprising, as fungi often respond to versatile environmental conditions by altering the type and yield of produced SMs (Brakhage, 2013). Interestingly, ISS-grown JSC-09335008 showed decreased production of all SMs, which is the opposite production pattern observed during the initial characterization of the metabolome of JSC-09335008 when compared to a “terrestrial” strain (Romsdahl et al., 2020). This discrepancy may be related to the fact that metabolomic profile of ISS-isolated JSC-09335008 was compared to the well-studied ATCC 1015 strain, rather than a “proper” JSC-09335008 ground control. Further investigation of the space environment-induced SM profile of the JSC-09335008 strain should be conducted to confirm whether decreased production of pestalamide B, nigerazine B (alkaloid), and nigragillin (alkaloid) are important biological adaptations. Given that sending experiments to the ISS is not readily available, it will be critical to use more easily accessible microgravity simulators, like High Aspect Ratio Vessels—HARV, or random positioning machines—RPMs to gain more insights in the space-induced phenotype of *A. niger* JSC-09335008 strain.

Finally, to gain insight into observed differences in acquired SMs profiles between current and previous study (Romsdahl et al., 2020) further experiment was conducted. Both ISS- and ground-grown JSC-09335008 were regrown in the same conditions (28°C for 5 days) as used in the previous study, which resulted in observing similar trends in SM production. After regrowing at 28°C, the ISS-grown JSC-09335008 produced higher yields of all SMs when compared to the regrown ground control, including approximately 60% increased production of the antioxidant pyranonigrin A. Pyranonigrin A was previously proposed to have a radioprotective nature, as pyranonigrin A-deficient JSC-09335008 strain was more sensitive to UVC exposure than the wild type JSC-09335008 strain (Romsdahl et al., 2020). Interestingly, pyranonigrin A production was not detected in the ISS- and ground-grown samples following the experiment on the ISS where growth temperature was about at 22°C. Due to this temperature-dependent discrepancy in observed SM profiles, the most important question to address is whether pyranonigrin A truly provides *A. niger* protection while in space. Such protection could potentially have various biotechnological applications for use of pyranonigrin A, including within human space programs and cancer therapies. Therefore, studies confirming pyranonigrin A potential as a radioprotective agent should be warranted. Finally, future studies should examine production of pyranonigrin A under various temperatures aboard the ISS, as well as its protective nature within the space environment to definitively answer this question.

This study is the first report of the multi-omics response of *A. niger* to space conditions during a controlled experiment, which enhances our understanding of its space-induced phenotype. Such understanding may be translated to development of protective measures for both astronauts and the spacecraft during future manned space explorations, as *A. niger* is ubiquitous fungus present in many human-occupied closed habitats (Checinska et al., 2015; Blachowicz et al., 2017). Lastly, a thorough understanding of the space-induced secondary metabolomic alterations of industrially important *A. niger* may result in creating a potent producer of compounds of interest during space voyages.

## Data Availability Statement

Raw WGS reads for JSC-093350089 ISS- and ground-grown are available in the NCBI SRA, under accession numbers SAMN25997338 and SAMN25997339 and BioProject accession number PRJNA807647. Proteomics data is accessible through Massive with the dataset identifier MSV000088986.

## Author Contributions

AB drafted the manuscript, contributed to sample processing, and conducted data analysis and interpretation. JR contributed to sample processing and data interpretation. AC and MK conducted protein sample processing, LC–MS analyses, and data processing. SM and JS contributed to genome analysis. TT designed the study and drafted the manuscript. KV and CW conceptualized the project, coordinated the flight experiment, designed the study, interpreted the data, and drafted the manuscript. All authors contributed to the article and approved the submitted version.

## Funding

This research was supported by the Center for the Advancement of Science in Space (CASIS) and sponsored by the International Space Station U.S. National Laboratory under grant/agreement number UA-2015-207 awarded to KV that funded a portion of the fellowship for AB. The funders had no role in study design, data collection and interpretation, the writing of the manuscript, or the decision to submit the work for publication.

## Conflict of Interest

The authors declare that the research was conducted in the absence of any commercial or financial relationships that could be construed as a potential conflict of interest.

The handling editor DT declared a past co-authorship with the authors AC, MK, JS, and KV.

## Publisher’s Note

All claims expressed in this article are solely those of the authors and do not necessarily represent those of their affiliated organizations, or those of the publisher, the editors and the reviewers. Any product that may be evaluated in this article, or claim that may be made by its manufacturer, is not guaranteed or endorsed by the publisher.

## Footnotes

^1^ https://broadinstitute.github.io/picard/
