# Supplementary material for: The International Space Station Environment Triggers Molecular Responses in Aspergillus niger
Source: Front Microbiol. 2022 Jun 30;13:893071. doi: 10.3389/fmicb.2022.893071 (PMC9280654; doi:10.3389/fmicb.2022.893071)
Supplement: Supplementary file 6 [file Table_6.DOCX]

Supplementary Material

The International Space Station environment triggers molecular responses in *Aspergillus niger*

Adriana Blachowicz^1,2^, Jillian Romsdahl^1^, Abby Chiang^3^, Sawyer Masonjones^4^, Markus Kalkum^3^, Jason E. Stajich^4^, Tamas Torok^5^, Clay C. C. Wang^1, 6^, and Kasthuri Venkateswaran^2*^

*** Correspondence:** Dr. Kasthuri Venkateswaran (Venkat): kjvenkat@jpl.nasa.gov

# Supplementary Figures and Tables

## Supplementary Figures

**
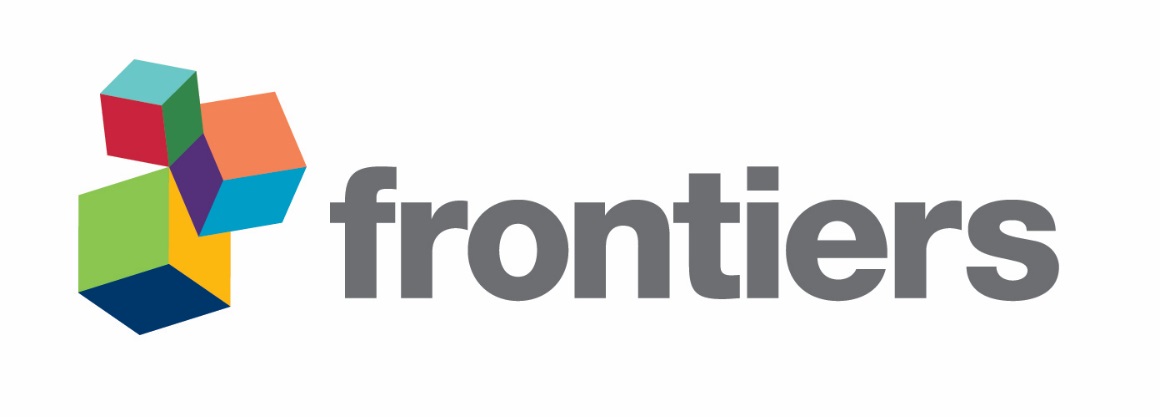
**

**Supplementary Figure 1. Project timeline and activities.** The overall length of the experiment was 42 days from a moment of preparing the samples by a science team to the splashdown and handover back to the science team for the follow up analyses; the active growth phase was 12 days at ~22°C. Beyond the active growth phase the samples were kept at 4°C. All activities for ground controls that were the exact mimics of the samples flown to the ISS were performed with roughly 2 h of delay with regard to the ISS samples.


**Supplementary Figure 2. The distribution of SNPs (A) and INDELS (B).** The pie charts present the occurrence and the distribution of the SNPs and INDELS throughout the *A. niger* genome

## Supplementary Tables

**Supplementary Table 1.** Identified Single Nucleotide polymorphisms (SNPs) when compared to CBS 513.88.

**Supplementary Table 2.** Identified INDELs when compared to CBS 513.88

**Supplementary Table 3.** Up-regulated proteins identified in the ISS-grown JSC-093350089 when compared to the ground control.

**Supplementary Table 4.** Down-regulated proteins identified in the ISS-grown JSC-093350089 when compared to the ground control.

**Supplementary Table 5.** Protein GO term enrichment analysis.
